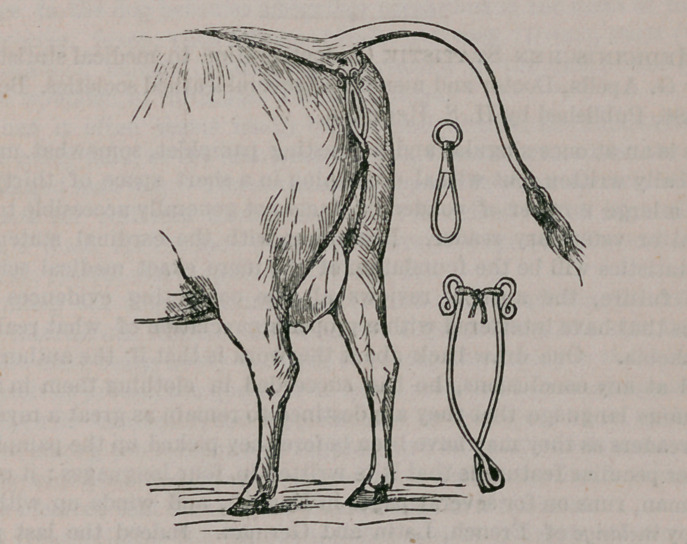# Progress of Veterinary Science

**Published:** 1886-10

**Authors:** 


### PROGRESS OF VETERINARY SCIENCE.

### PURE PEPSIN.

(Abstract from *American Analyst.*)

The United States Pharmacœpia recognizes saccharated pepsin only—this is, pepsin containing ninety per cent. of sugar of milk to every ten per cent. of actual pepsin. It will therefore, be readily seen that if a pure pepsin, that is an undiluted or concentrated pepsin, could be manufactured it would be more than ten times as strong as either the saccharated or amyl pepsin. Such has been made, and is known variously as concentrated, crystallized and scale pepsin.

Physicians when prescribing any remedy naturally desire to use that which is most reliable, even in strength, and one always to be depended upon ; hence when they have once decided which preparation they will use in their prescriptions, they want to be certain that that particular preparation is the one used by the apothecary, and therefore, they should be careful to add the name of the manufacturer whose goods they wish, and not trust to the simple word "pepsin," or any of the objectives applied to it by different manufacturers, for the reason that many apothecaries will take advantage of these open doors to use the cheapest pepsin they can buy, and the result to the patient will be different from what the physician had a right to expect, greatly to his disappointment and injury to his reputation. There is no doubt that, like all good things, the best pepsin made has been imitated.

*American Analyst* tested twelve samples of pepsin manafactured by six different firms. All of these were obtained at drug stores, and, after being emptied into clean glass bottles, were numbered from one to twelve, and handed to two different chemists without any knowledge on their part as to the maker of any of the samples submitted. Their reports are as follows:

Number of grains of egg albumen in finely pulverized form dissolved by two grains of pepsin in six hours at a temperature 100–103° F.:

| | |
|---|---|
| Sample No. 1 | 18 grains. |
| " " 2 | 19.7 " |
| " " 3 | inert. |
| " " 4 | " |
| " " 5 | 508 grains. |
| " " 6 | 506 " |
| " " 7 | 2018 " |
| " " 8 | 2007 " |
| " " 9 | 174 " |
| " " 10 | 336 " |

It is fair to add that specimens Nos. 7 and 8 were Carl Jensen's crystal pepsin.

The largest wholesale drug house in the world, that of Gehe & Co., in Dresden, Germany, have placed the weight of their influence on the side of Jensen's by buying his product to the exclusion of every other.

PASTEURIANA.—A new form of religion has sprung up, of which Pasteur is the high priest, and in which we may say the devil is represented by the virus of rabies. The cult is taken up by young enthusiasts and middle aged men, with perfervid imaginations. They have touched the hem of Pasteur's garment, they have made the pilgrimage to Paris, they have seen the little harmless injections performed, and they are believers. Reason, facts, etc., are thrown on one side. We shall not attempt to disturb their faith, for in this connection science and faith are distinct things, and we do not argue with "Faith Healers." The latest phase of this cult is extraordinary. It is no longer a question of a sure and certain remedy for rabies. It has come down to the law of averages. Pasteur's results are better than those of any previous or contemporary hydrophobia curer—*voila tout.* Dealing with a poison or virus, which acts no one knows how, with a cultivated fluid, which also acts no one knows how, *empirical results* are all we have to depend on. This is misnamed scientific evidence. We admire the simplicity of character of these, who accept such scientific evidence, and who are so indignant at any question of doubt, etc. In *Progres Med.*, July 10th, 1886, page 586, we read, "Elvina Lagut, aged 11, was bitten at Chassagne (Jura), on 27th April last. She was brought to the laboratory of the Ecole Normale *nine days* after. During the fifteen days she passed at Paris she followed the prescribed treatment with the greatest regularity, and received the ten progressive *bouillons.* She was *declared cured* and sent back to her family. On the 13th June this child presented the first symptoms of rabies, dying on 17th June." We regret we cannot join in the chorus of satisfaction at the results obtained by M. Pasteur, in face of such results as the above. Without antagonism our stand is on the motto—

"Pax et scientia, sed veritas sine timore."

[*Provincial Med. Journal.*

HYDROPHOBIA—It has recently come to light, that the State of New York, in 1806, paid to John M. Crous a thousand dollars for a remedy against hydrophobia which he considered infallible. This measure was advocated by DeWitt Clinton and Chancellor Kent. This remedy consisted of one ounce of the jawbone of a dog, burnt and pulverized; the false tongue of a newly foaled colt, dried and pulverized; a scruple of verdigris, raised on the surface of old copper by laying it in moist earth. The warrant of the Comptroller on which the money was paid, and the receipt of Crous, are on file with other state papers at Albany.—*Science.*

A NEW CONSTRICTOR OF THE VULVA.—Of the numerous retentive bandages and ingenious belts designed for the relief of *prolapsus uteri* and *prolapsus vagina*, preference has been given to the apparatus of Delwart, with which veterinarians are now well familiar. Nevertheless, this admirable contrivance has its inconveniences. The bands enclosing the

*labia majora* become easily displaced, and are difficult to graduate to the varying tumefaction, besides, they absorb the urine and become filthy, putrefaction taking place in the tissues beneath, while edema and gangrene often result. Recognizing these drawbacks, Lund devised the simple iron triangle so warmly recommended by Lanzellotti-Buonsanti, Saint Cyr and others. This also had its disadvantages, in that it did not adapt itself to the varying dimensions of the vulva, and that the upper end of the triangle projected too much or too little, according to the height of the tail.

To modify, therefore, this ingenious instrument of Lund, render it equally applicable to every size vulva, shape it to their ovoid form, make it possible to lengthen, shorten, widen, etc., when necessary, be covered with tow or have same removed without changing the cord, and render the constriction at the same time elastic and powerful without making it difficult to apply or costly in price, and without sacrificing the primitive admirable simplicity of the iron was my intention and, it appears to me, I have succeeded.

The instrument is constructed of a simple iron wire of about the thickness of a goose-quill, 70 centimeters long. The wire held firmly at the center is made to take two turns on itself, so as to make a ring about the size of the little finger. The wires on either side are then bent, forming an acute angle at the point of divergence, and the extremities folded, first outwards, then inwards on itself so as to form a figure 8. The ends converge at this point, giving the spring an ovoid shape, besides a curve is given to it so that when resting on a plane surface, it is sustained by two corresponding points of the branches working like a ship. I insist on this curvature. It greatly contributes to the easy adaptation of the instrument, and once in position to its perfect immobility. To the lower ring of the instrument a snap is fixed, and through the ring of this a cord is passed. Cords are also attached to the lateral rings.

The iron being placed on the vulvar region so that the two branches embrace the *labia majora*, the two upper cords are crossed over the lumbar region and tied, the left one to the right side of the belt and *vice versa*. The lower cords are passed one along the right inguinal region, the other along the left, and attached to the same belt a little lower down. Then a strong twine is passed through the two upper lateral rings and tied, causing the branches to approximate more or less as required. The advantage of the snap is in the facility it affords for changing the two. The materials are cheap and any blacksmith can make half a dozen of these instruments in two hours. *Eugenio Aruch V. S., in La Clinica Veterinaria.* Pisa, February, 1886.

## Figures and Tables

**Figure f1:**